# Higher remnant cholesterol increases the risk of coronary heart disease and diabetes in postmenopausal women

**DOI:** 10.3389/fendo.2024.1475933

**Published:** 2024-12-09

**Authors:** Yan Zhang, Kexin Song, Shuli Bi, Mingyang Li, Zhuhua Yao

**Affiliations:** ^1^ Tianjin Union Medical Center, Tianjin Medical University, Tianjin, China; ^2^ Department of Internal Medicine, Graduate School of Hebei Medical University, Shijiazhuang, China; ^3^ School of Medicine, Nankai University, Tianjin, China; ^4^ Clinical School of Thoracic, Tianjin Medical University, Tianjin, China; ^5^ The Institute of Translational Medicine, Tianjin Union Medical Center of Nankai University, Tianjin, China; ^6^ Department of Cardiology, Tianjin Union Medical Center, Tianjin, China

**Keywords:** remnant cholesterol, coronary heart disease, diabetes, postmenopausal women, risk factor

## Abstract

**Background:**

Postmenopausal women represent the demographic increasingly susceptible to cardiovascular and metabolic diseases. Elevated levels of remnant cholesterol (RC) have been implicated in atherosclerosis and insulin resistance.

**Methods:**

This study aimed to investigate the relationship between RC and the prevalence of coronary heart disease (CHD), diabetes, and CHD combined with diabetes in a nationally representative sample of US postmenopausal women using data from the National Health and Nutrition Examination Survey (NHANES) 2007-2018. Multivariate logistic regression models were employed to evaluate the association between RC and the outcomes of interest. Nonlinear associations were assessed using restricted cubic splines (RCS), and subgroup analyses, along with interaction tests, were performed.

**Results:**

A total of 1611 participants were included in the final analysis. Higher RC levels were significantly associated with increased risks of CHD [OR=1.67, 95%CI (1.02, 2.74)], diabetes [OR=1.77, 95%CI (1.22, 2.58)], and CHD combined with diabetes [OR=2.28, 95%CI (1.17, 4.42)] (all P<0.05). Compared to the lowest RC quartile (Q1), the highest quartile (Q4) demonstrated elevated incidences of CHD [OR=1.76, 95%CI (1.04, 2.98)], diabetes [OR=1.81, 95%CI (1.30, 2.53)], and CHD combined with diabetes [OR=3.08, 95%CI (1.29, 7.37)] (all P<0.05). RCS curves indicated a nearly linear relationship between RC and the risks of CHD, diabetes, and CHD combined with diabetes.

**Conclusion:**

Our study reveals a significant positive correlation between RC levels and the prevalence of CHD, diabetes, and CHD combined with diabetes among postmenopausal women. Understanding these associations could potentially inform targeted prevention and management strategies tailored to this vulnerable population.

## 1 Introduction

Cardiometabolic diseases (CMD) encompass a spectrum of chronic non-communicable conditions intricately linked to metabolic and cardiovascular health, prominently characterized by dyslipidemia and clustering of other metabolic risk factors (1, 2). CMD, such as cardiovascular diseases (CVD) (3) and diabetes (4) exhibit significant alterations in circulating lipoprotein profiles. Globally, diabetes and coronary heart disease (CHD) represent leading causes of mortality and disability, imposing substantial public health burdens (5, 6). CHD frequently coexists with diabetes, likely due to shared risk factors between these conditions. For instance, diabetic patients often present with an atherogenic lipid profile, including abnormalities in blood lipids and lipoproteins (7). Abnormalities in lipid metabolism not only constitute well-established risk factors for CHD but also correlate with pancreatic β-cell dysfunction and insulin resistance (IR), contributing to the pathogenesis of diabetes (8). Lowering plasma low-density lipoprotein cholesterol (LDL-C) remains a cornerstone preventive strategy for CHD, as strongly recommended by current guidelines (9). However, despite achieving recommended LDL-C levels, recurrence rates of cardiovascular events remain notably high, underscoring a substantial residual risk

In recent years, remnant cholesterol (RC) has emerged as a pivotal marker of lipid abnormalities resistant to statin therapy (10). Increasing evidence has underscored the association between elevated RC levels and heightened risk as well as adverse outcomes of CHD and diabetes (11, 12). RC denotes the cholesterol content within triglyceride-rich lipoproteins (TRLs), encompassing very low-density lipoproteins (VLDL), intermediate-density lipoproteins (IDL), and chylomicron remnants (CR) (13). While plasma triglyceride (TG) levels can serve as a clinical surrogate for RC, RC exerts a more direct impact on cardiovascular disease (14), contributing to atherosclerosis through mechanisms such as direct accumulation in arterial walls and enhanced inflammatory responses (15). Furthermore, individuals with elevated RC levels are predisposed to metabolic disorders such as diabetes and metabolic syndrome (16). The association between RC and diabetes risk has been robustly established across diverse populations (8, 17).

Menopause exerts profound impacts on the social, physiological, and psychological health of women. Postmenopausal women typically exhibit a lipid profile that is considered disadvantageous for health compared with premenopausal women, characterized by elevated levels of LDL-C, TG, and total cholesterol (TC) (18). Epidemiological studies indicate that while the incidence of CHD in premenopausal women is approximately half that of age-matched men, this disparity diminishes following menopause (19). The decline in ovarian function and hormonal imbalance during menopause can promote abdominal obesity and central visceral fat accumulation, a pattern linked to insulin resistance in non-adipose tissues and organs, significantly increasing the risk of diabetes among postmenopausal women (20). Given these contributing factors and heightened risks, there is an urgent need for increased attention and proactive management of postmenopausal women. Therefore, this study aims to utilize nationwide large-scale data to investigate the association between non-traditional lipid parameter RC and the incidence of coronary heart disease and diabetes in this specific population.

## 2 Materials and methods

### 2.1 Study population

**Figure 1:**
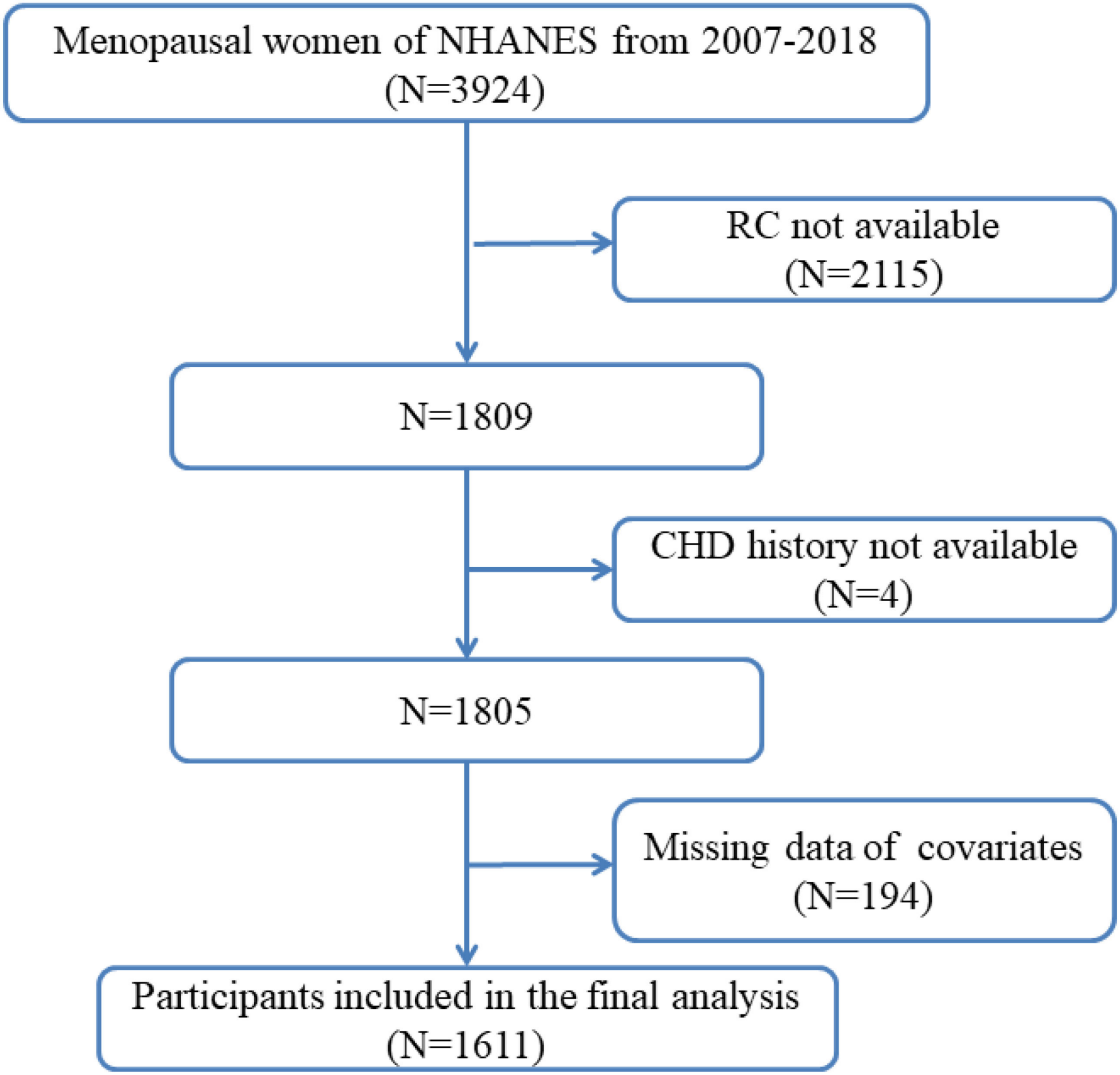
Flow chart of participants selection from the National Health and Nutrition Examination Survey (NHANES) 2007–2018.

The National Health and Nutrition Examination Survey (NHANES) is an ongoing series of surveys designed to evaluate the health and nutritional status of both adults and children in the United States. These surveys involve comprehensive data collection through interviews and physical examinations. Approval for NHANES protocols is obtained from the Institutional Review Board of the National Center for Health Statistics (NCHS), and informed consent is obtained from all participants. All procedures were conducted in accordance with the principles outlined in the Helsinki Declaration. Additional detailed information about NHANES is available on their official website. For this study, data from NHANES spanning 2007 to 2018 were abstracted. The study focused on utilizing reproductive health information to determine menopausal status among participants. Inclusion criteria encompassed women classified as postmenopausal or experiencing a cessation of menstruation within the past 12 months due to lifestyle changes. Participants with incomplete or unknown data were excluded from the analysis, resulting in a final cohort of 1611 postmenopausal women included in the study (**Figure 1**).

### 2.2 Data collection

Participant data encompassed demographic details, laboratory test results, and medical conditions. Demographic information comprised age, ethnicity, educational attainment, marital status, smoking status, height, and weight. Educational levels were categorized as less than high school, high school, and more than high school. Marital status was delineated as either without a partner or with a partner. Laboratory tests included measurements of fasting blood glucose (FBG), HbA1c, TC, TG, LDL-C, and high-density lipoprotein cholesterol (HDL-C). Medical information pertaining to the diagnosis or treatment of hypertension, diabetes, and CHD, as well as reproductive health data, was also collected.

### 2.3 Definitions

RC was calculated using the following formula: RC (mmol/L) = TC (mmol/L) -LDL-C (mmol/L) - HDL-C (mmol/L) (21). Body mass index (BMI) was calculated as weight (kg) divided by height (m) squared. Smoking at least 100 cigarettes in a lifetime was defined as a smoker (22). Hypertension was defined as self-reported diagnosis or current use of antihypertensive medication. Diabetic status was defined as self-reported diagnosis, use of hypoglycemic agents or insulin, FBG≥7mmol/L, or HbA1c≥6.5% (23). CHD was defined as self-reported diagnosis of CHD, myocardial infarction (MI), or angina pectoris (24).

### 2.4 Statistical analysis

Baseline characteristics of the study population were stratified according to RC quartiles. Continuous variables were expressed as mean ± standard deviation (SD) and compared using one-way analysis of variance (ANOVA), while categorical variables were presented as numbers (percentages) and compared using the chi-square test. Bivariate associations between RC and continuous baseline variables were examined using Pearson correlation analysis. Differences in RC levels between groups were assessed using independent-sample t-tests. The relationship between RC levels and various outcomes (CHD, diabetes, and combined CHD with diabetes) was evaluated using multivariate logistic regression analysis, presenting odds ratios (ORs) and 95% confidence intervals (CIs) across different models. Adjustments were made for covariates such as age, ethnicity, education level, marital status, BMI, smoking status, hypertension, diabetes, and CHD. Sensitivity analysis was conducted by categorizing RC into quartiles to validate the robustness of the findings. Restricted cubic splines (RCS) curves were employed to explore potential non-linear associations between RC and the various outcomes. Subgroup analyses and interaction tests were performed using multivariate logistic regression, stratified by age, BMI, smoking status, hypertension, and diabetes/CHD status. Statistical analyses were performed using SPSS 25.0 (IBM, Armonk, New York, USA) and R (version 4.2). A significance level of P < 0.05 was considered statistically significant.

## 3 Results

### 3.1 Baseline characteristics

**Figure 2:**
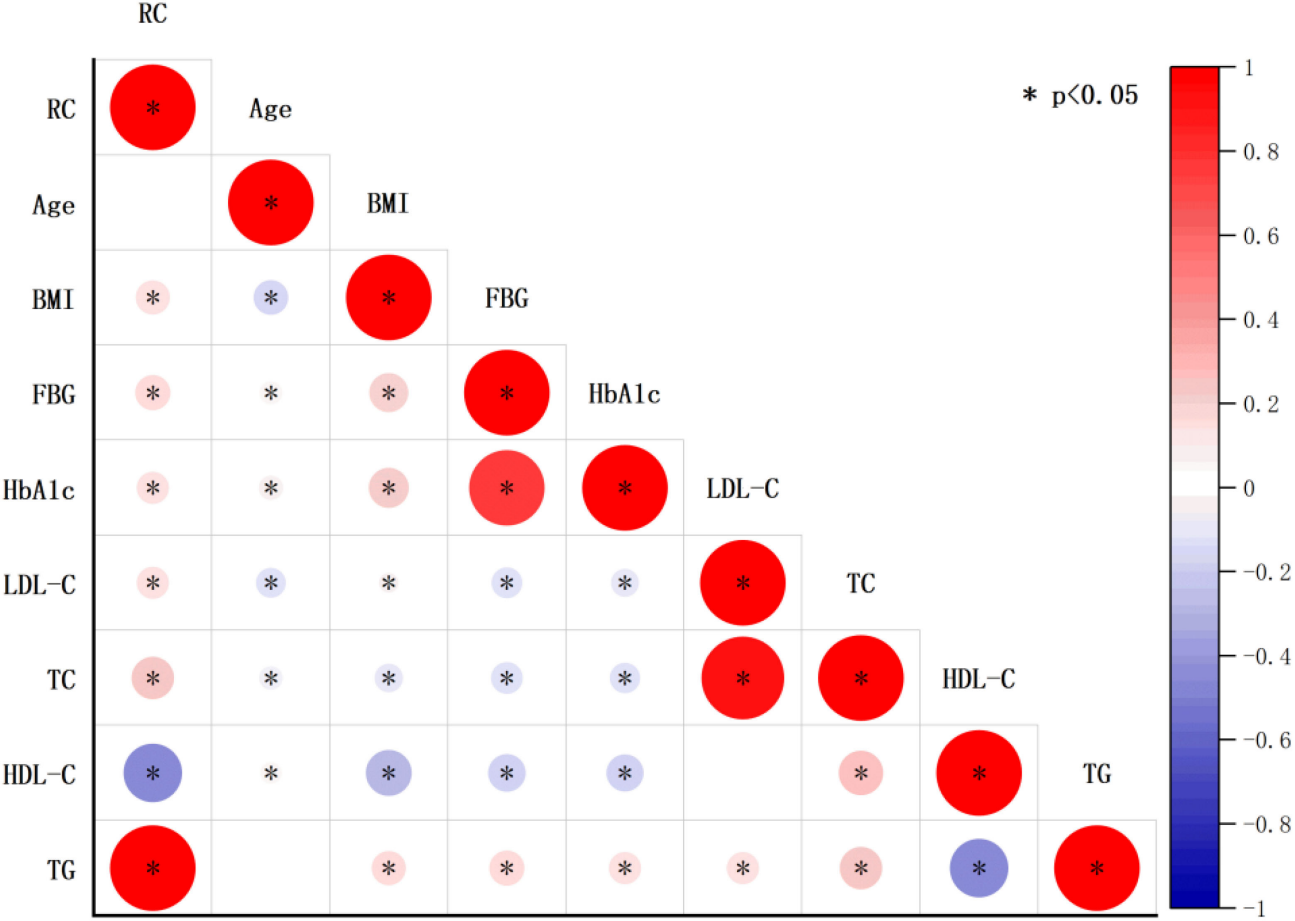
The correlations between RC and baseline continuous variables.

**Figure 3:**
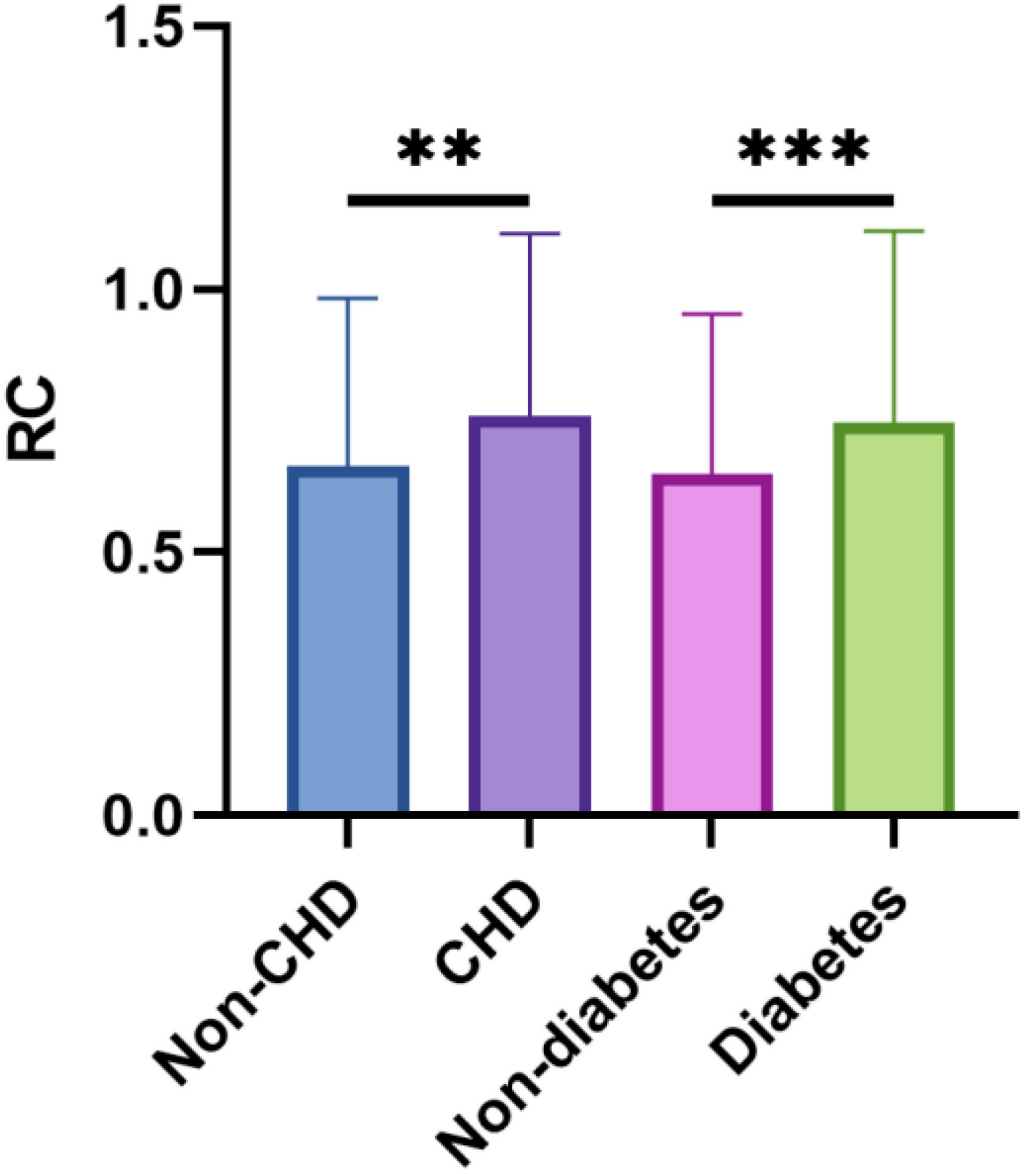
The RC levels between different groups. **p < 0.01, ***p < 0.001.

A total of 1611 postmenopausal women who met the inclusion criteria were included in the final analysis. **Table 1** presents the baseline characteristics of the study participants, stratified by RC quartiles. Significant differences were observed across various parameters including ethnicity, educational level, marital status, BMI, presence of diabetes and CHD, FBG, HbA1c, TC, TG, LDL-C, and HDL-C. Participants in the highest RC quartile were more likely to be non-Hispanic white, have lower educational attainment (less than high school), be partnered, and smoke. Additionally, this group had a higher prevalence of hypertension, diabetes, and CHD, along with higher BMI, FBG, HbA1c, TC, TG, and LDL-C levels, and lower HDL-C levels. The correlations between RC and baseline continuous variables using Pearson correlation analysis were shown in **Figure 2**. RC showed positive correlations with BMI, FBG, HbA1c, LDL-C, TC, and TG, while demonstrating a negative correlation with HDL-C (P < 0.05). **Figure 3** depicts the distribution of RC levels across different groups. Participants with both CHD and diabetes tended to exhibit higher RC levels.

**Table 1:** Characteristics of the study population based on RC quartiles.

Variables	RC Quartiles				
	Q1(n=403)	Q2(401)	Q3(404)	Q4(403)	P-Value
**Age (years)**	63.35 ± 10.75	64.21 ± 10.83	65.11 ± 10.77	64.12 ± 10.86	0.148
**Ethnicity n (%)**					<0.001
Mexican American	27 (6.70)	50 (12.47)	55 (13.61)	63 (15.63)	
Non-Hispanic White	191 (47.39)	202 (50.37)	211 (52.23)	228 (56.58)	
Non-Hispanic Black	138 (34.24)	83 (20.70)	61 (15.10)	38 (9.43)	
Other Hispanic	28 (6.95)	38 (9.48)	55 (13.61)	51 (12.66)	
Other Race	19 (4.71)	28 (6.98)	22 (5.45)	23 (5.71)	
**Educational level n (%)**					<0.001
Less than high school	83 (20.60)	107 (26.68)	112 (27.72)	155 (38.46)	
High school	95 (23.57)	111 (27.68)	104 (25.74)	110 (27.30)	
More than high school	225 (55.83)	183 (45.64)	188 (46.53)	138 (34.24)	
**Marital status n (%)**					0.049
Without partner	221 (54.84)	210 (52.37)	185 (45.79)	195 (48.39)	
Having a partner	182 (45.16)	191 (47.63)	219 (54.21)	208 (51.61)	
**BMI (kg/m2)**	27.29 ± 6.26	27.84 ± 6.20	29.71 ± 6.37	29.96 ± 5.90	<0.001
**RC (mmol/L)**	0.35 ± 0.06	0.51 ± 0.05	0.71 ± 0.07	1.13 ± 0.26	<0.001
**Smoker n (%)**					0.106
Yes	162 (40.20)	147 (36.66)	157 (38.86)	181 (44.91)	
NO	241 (59.80)	254 (63.34)	247 (61.14)	222 (55.09)	
**Hypertension n (%)**					0.051
Yes	219 (54.34)	219 (54.61)	248 (61.39)	247 (61.29)	
NO	184 (45.66)	182 (45.39)	156 (38.61)	156 (38.71)	
**Diabetes n (%)**					<0.001
Yes	80 (19.85)	89 (22.19)	109 (26.98)	129 (32.01)	
NO	323 (80.15)	312 (77.81)	295 (73.02)	274 (67.99)	
**CHD n (%)**					0.019
Yes	25 (6.20)	38 (9.48)	42 (10.40)	51 (12.66)	
NO	378 (93.80)	363 (90.52)	362 (89.60)	352 (87.34)	
**FBG (mmol/L)**	5.83 ± 1.45	5.98 ± 1.58	6.30 ± 1.99	6.46 ± 2.07	<0.001
**HbA1c (%)**	5.77 ± 0.73	5.89 ± 0.96	6.05 ± 1.11	6.08 ± 1.01	<0.001
**TC (mmol/L)**	5.11 ± 0.98	5.24 ± 1.06	5.32 ± 1.00	5.81 ± 1.13	<0.001
**TG (mmol/L)**	0.76 ± 0.14	1.12 ± 0.11	1.54 ± 0.15	2.46 ± 0.57	<0.001
**LDL-C (mmol/L)**	2.95 ± 0.80	3.10 ± 0.94	3.18 ± 0.92	3.40 ± 1.06	<0.001
**HDL-C (mmol/L)**	1.81 ± 0.44	1.63 ± 0.38	1.44 ± 0.31	1.28 ± 0.30	<0.001

Continuous data are presented as mean ± standard deviation (SD). Categorical data are presented as frequencies (%).BMI, body mass index; RC, remnant cholesterol; CHD, coronary heart disease; FBG, fasting blood glucose; TC, total cholesterol; TG, triglyceride; LDL-C, low-density lipoprotein cholesterol; HDL-C, high-density lipoprotein cholesterol.

### 3.2 Association between RC and CHD

The association between RC and CHD was displayed in **Table 2**. According to the findings of our study, there was a positive link between RC and CHD in both Model1 [OR=2.25, 95%CI (1.43, 3.56)] and model2 [OR=2.10, 95%CI (1.30, 3.39)]. After fully adjusting for covariates (Model 3), RC remained markedly positively correlated with CHD [OR=1.67, 95%CI (1.02, 2.74)]. In order to perform a sensitivity analysis, RC was divided into quartiles and the OR for Q1, Q2, Q3, and Q4 in model 3 were 1.00, 1.51(0.88, 2.60), 1.53(0.89, 2.62), and 1.76(1.04, 2.98), respectively. Compared to Quartile 1, participants in Quartile 4 showed a 1.76-fold increase in the incidence of CHD (p for trend > 0.05 in model3 while < 0.05 in model1 and 2).

**Table 2:** Multivariate logistic regression analysis of association between RC and the risk of coronary heart disease.

	Model 1	Model 2	Model 3
	OR (95%CI)P-Value	OR (95%CI)P-Value	OR (95%CI)P-Value
RC	2.25 (1.43, 3.56) ***	2.10 (1.30, 3.39) **	1.67 (1.02,2.74)*
RC (quartiles)			
Q1	Reference	Reference	Reference
Q2	1.58 (0.94,2.68)	1.49 (0.88,2.54)	1.51 (0.88,2.60)
Q3	1.75 (1.05,2.94)*	1.63 (0.96,2.75)	1.53 (0.89,2.62)
Q4	2.19 (1.33,3.61)**	1.98 (1.18,3.31)**	1.76 (1.04,2.98)*
P for trend	0.003	0.014	0.069

Model 1: no adjustment for any variables.

Model 2: adjusted for age, ethnicity, education level and marital status.

Model 3: adjusted for Model 2 plus BMI, smoking status, hypertension, diabetes.

OR, odds ratios; CI, confidence interval, *p < 0.05, **p < 0.01, ***p < 0.001.

### 3.3 Association between RC and diabetes

**Table 3** presents the association between RC and diabetes. Our analysis revealed a consistent positive relationship between RC levels and diabetes across various models. In Model 1, OR was 2.38 (95% CI 1.71, 3.33), while in Model 2, it was 2.32 (95% CI 1.64, 3.28). After comprehensive adjustment for covariates in Model 3, RC remained significantly associated with diabetes, with an OR of 1.77 (95% CI 1.22, 2.58). In Model 2, compared to Quartile 1 (reference), Quartile 4 exhibited a 1.81-fold increased risk of diabetes, with OR of 1.81 (95% CI 1.30, 2.53). The trend analysis across quartiles showed statistically significant trends (all p for trend < 0.05), emphasizing a dose-response relationship.

**Table 3:** Multivariate logistic regression analysis of association between RC and the risk of diabetes.

	Model 1	Model 2	Model 3
	OR (95%CI)P-Value	OR (95%CI)P-Value	OR (95%CI)P-Value
RC	2.38 (1.71, 3.33)***	2.32 (1.64, 3.28) ***	1.77 (1.22,2.58) **
RC (quartiles)			
Q1	Reference	Reference	Reference
Q2	1.15 (0.82,1.62)	1.12 (0.79,1.58)	1.03 (0.71,1.49)
Q3	1.49 (1.07,2.07)*	1.49 (1.06,2.09)*	1.14 (0.79,1.64)
Q4	1.90 (1.38,2.62)***	1.81 (1.30,2.53)***	1.40 (0.98,2.01)
P for trend	<0.001	<0.001	0.036

Model 1: no adjustment for any variables.

Model 2: adjusted for age, ethnicity, education level and marital status.

Model 3: adjusted for Model 2 plus BMI, smoking status, hypertension, coronary heart disease.

OR, odds ratios; CI, confidence interval, *p < 0.05, **p < 0.01, ***p < 0.001.

### 3.4 Association between RC and CHD combined with diabetes

AS shown in **Table 4**, there was also a positive link between RC and CHD combined with diabetes in Model1 [OR=2.89, 95%CI (1.57, 5.33)], model2 [OR=2.67, 95%CI (1.40, 5.09)] and model 3 [OR=2.28, 95%CI (1.17, 4.42)] respectively. The OR for Q1, Q2, Q3, and Q4 in model 3 were 1.00, 2.35(0.96, 5.79), 2.57(1.06, 6.22), and 3.08(1.29, 7.37), respectively. Compared to Quartile 1, participants in Quartile 4 showed a 3.08-fold increase in the incidence of CHD combined with diabetes (all p for trend < 0.05).

**Table 4:** Multivariate logistic regression analysis of association between RC and the risk of coronary heart disease combined with diabetes.

	Model 1	Model 2	Model 3
	OR (95%CI)P-Value	OR (95%CI)P-Value	OR (95%CI)P-Value
RC	2.89 (1.57, 5.33)***	2.67 (1.40, 5.09) **	2.28 (1.17,4.42) *
RC (quartiles)			
Q1	Reference	Reference	Reference
Q2	2.66 (1.10,6.44)*	2.48 (1.02,6.06)*	2.35 (0.96,5.79)
Q3	3.26 (1.38,7.72)**	2.97 (1.24,7.11)*	2.57 (1.06,6.22)*
Q4	4.06 (1.75,9.44)**	3.56 (1.51,8.41)**	3.08 (1.29,7.37)*
P for trend	0.002	0.007	0.026

Model 1: no adjustment for any variables

Model 2: adjusted for age, ethnicity, education level and marital status.

Model 3: adjusted for Model 2 plus BMI, smoking status, hypertension.

OR, odds ratios; CI, confidence interval, *p < 0.05, **p < 0.01, ***p < 0.001.

### 3.5 RCS analysis

**Figure 4:**
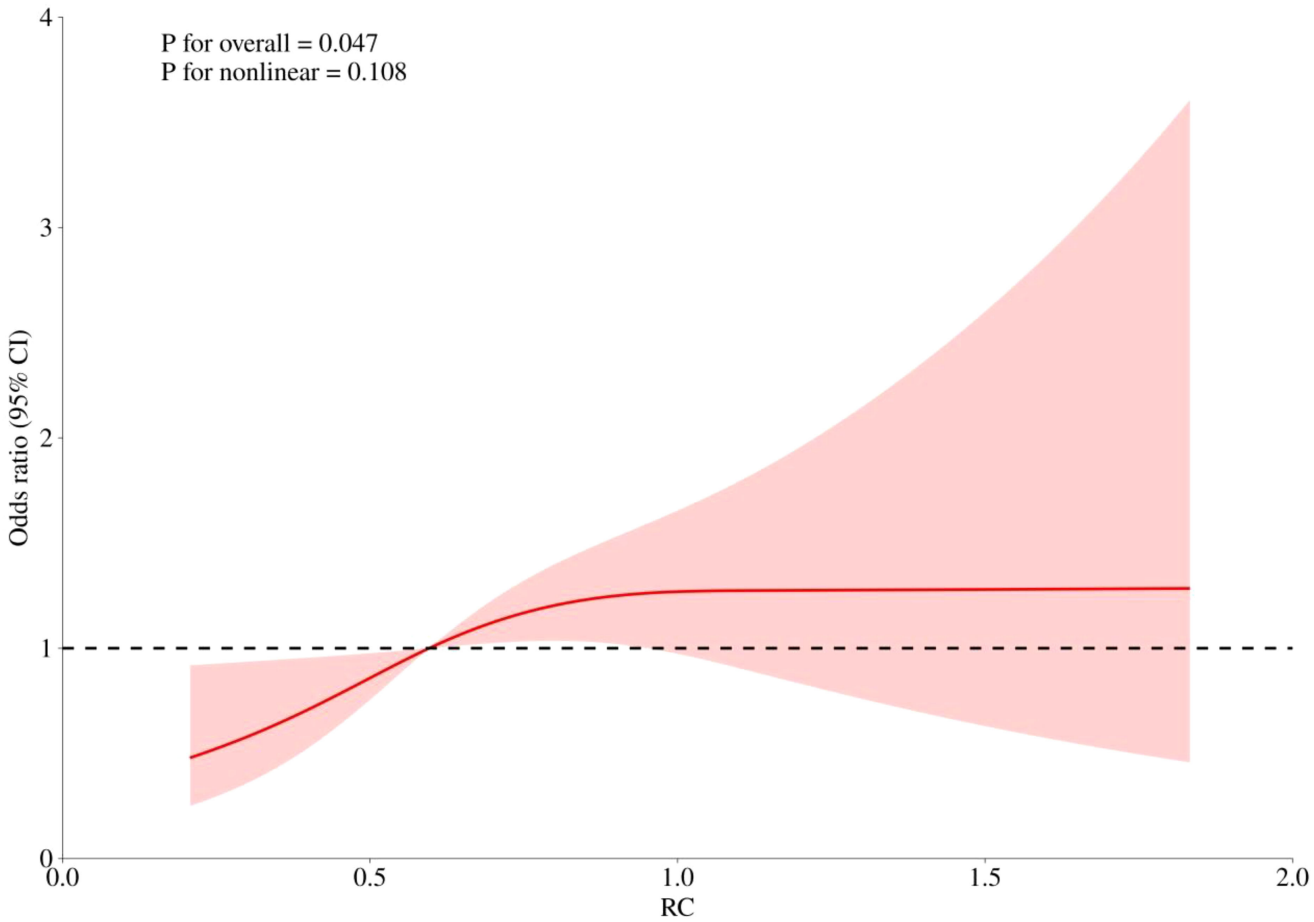
The RCS analysis between RC and risk of CHD.

**Figure 5:**
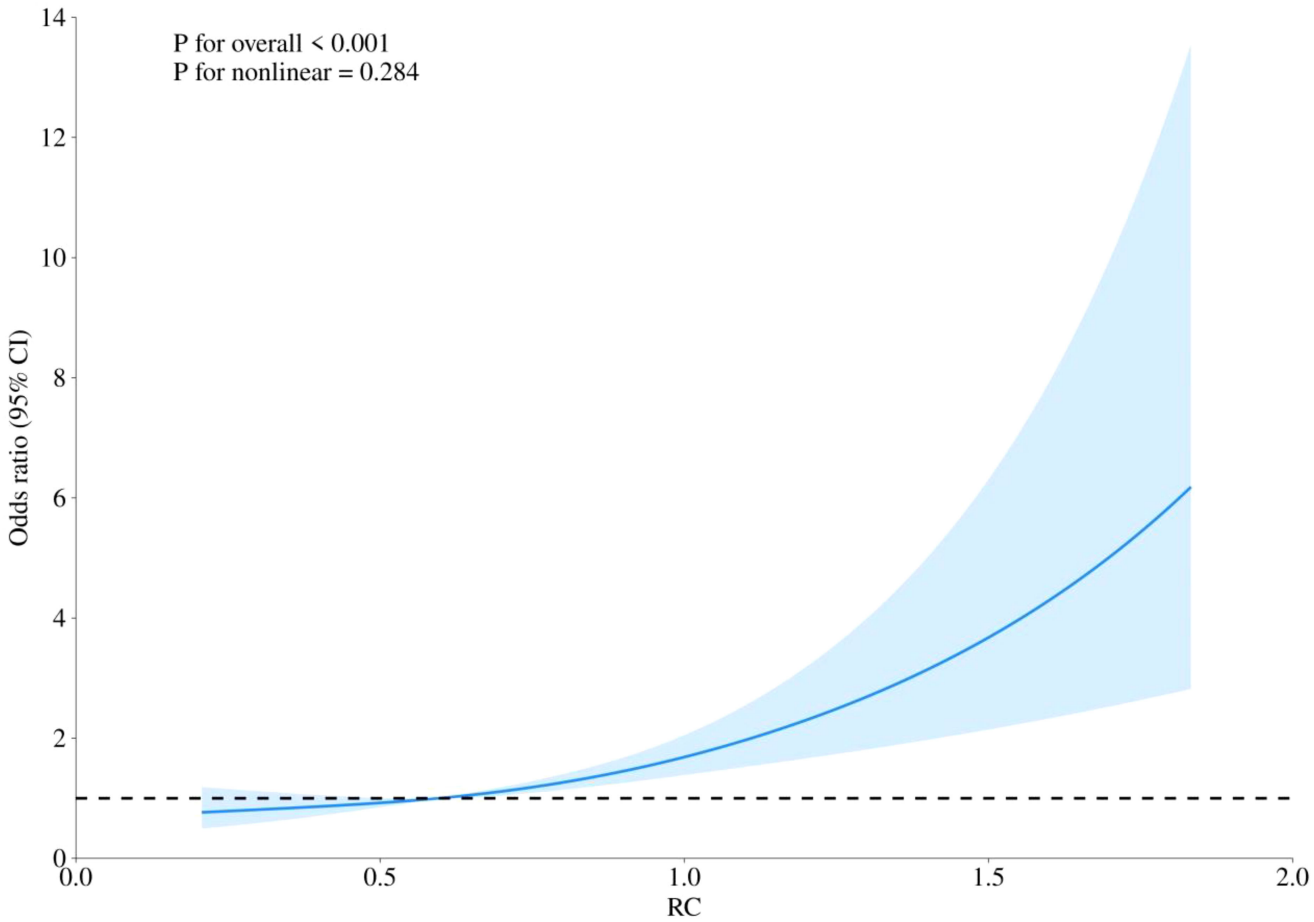
The RCS analysis between RC and risk of diabetes.

**Figure 6:**
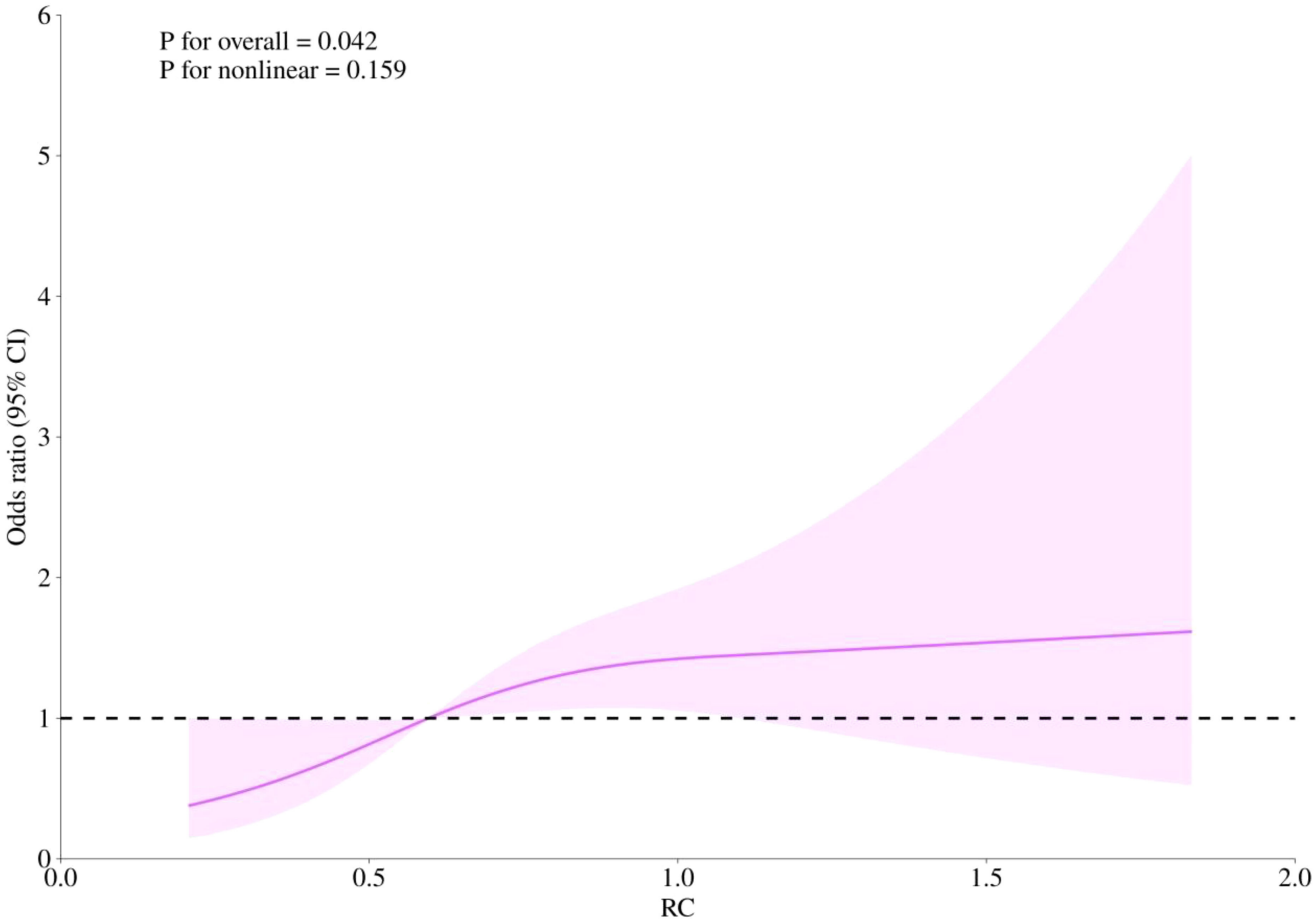
The RCS analysis between RC and risk of CHD combined with diabetes.

RCS curves were employed to examine potential nonlinearity in the association between RC levels and the risks of CHD, diabetes, and the combined outcome of CHD with diabetes, as depicted in **Figures 4**–**6**. Our findings indicated predominantly linear relationships between RC levels and the risk of CHD (P for overall trend = 0.047, P for nonlinearity = 0.108), diabetes (P for overall trend < 0.0001, P for nonlinearity = 0.284), and CHD combined with diabetes (P for overall trend = 0.042, P for nonlinearity = 0.159).

### 3.6 Subgroup analysis

**Figure 7:**
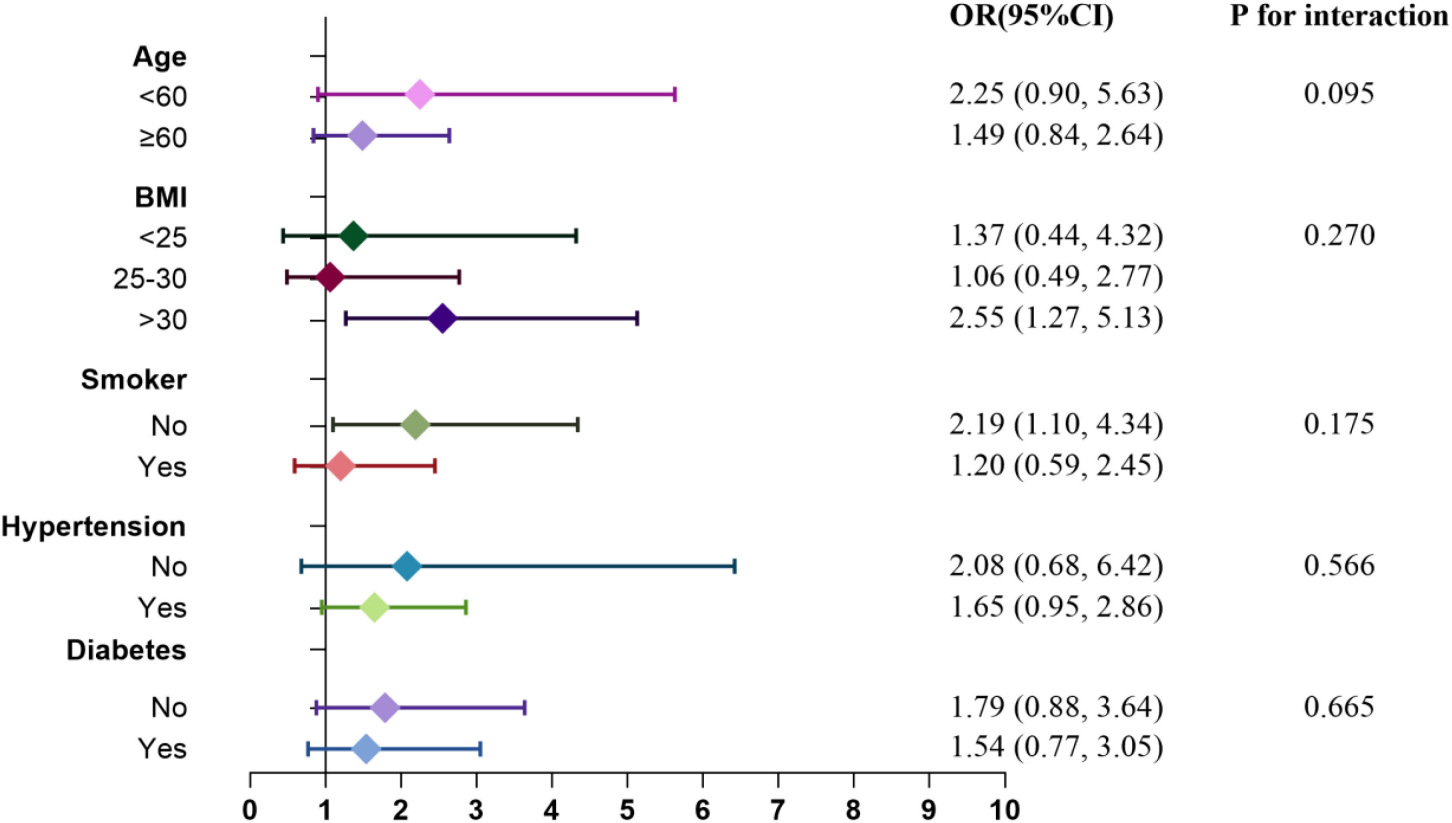
Subgroup analysis for the association between RC and risk of CHD.

**Figure 8:**
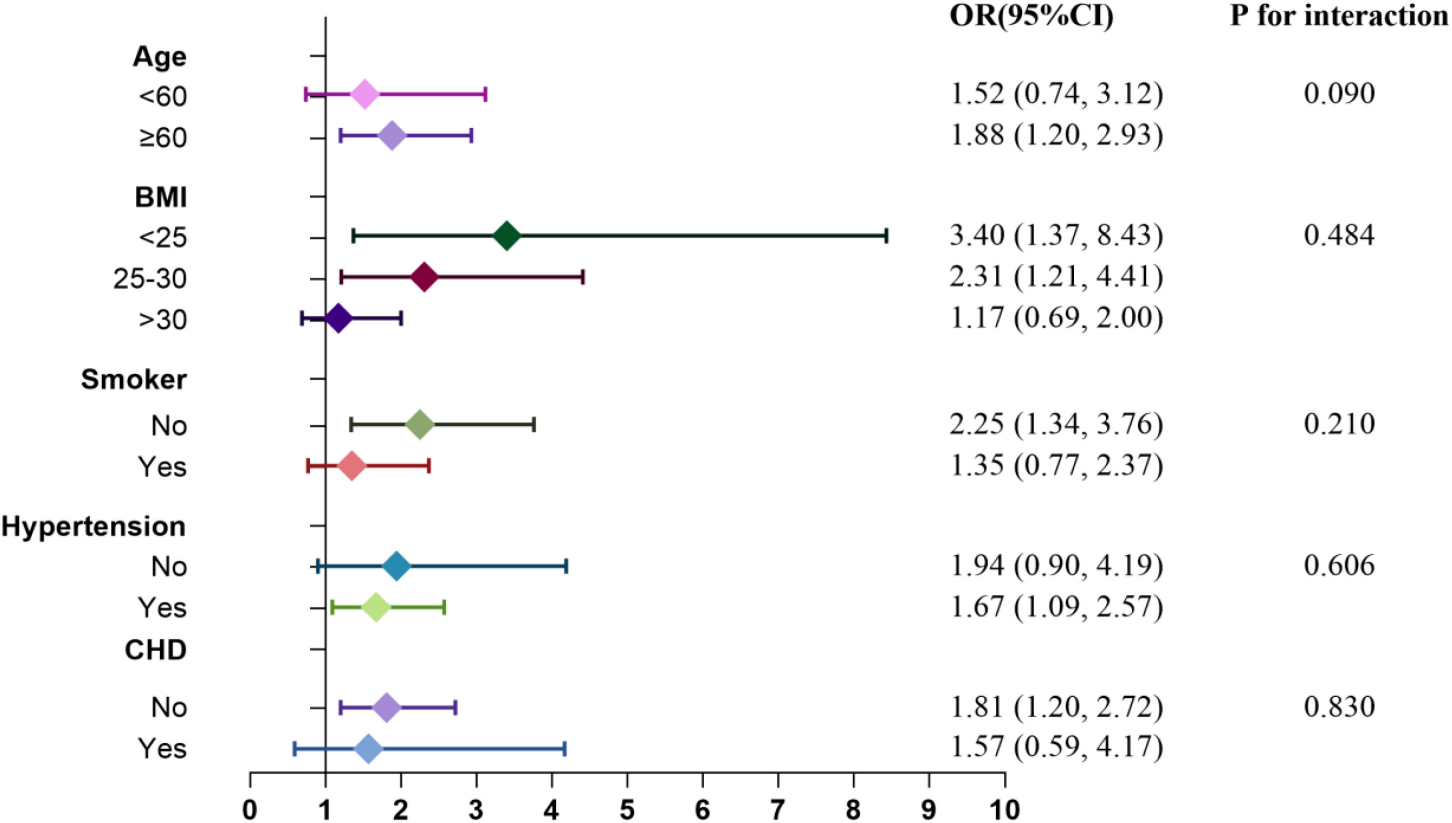
Subgroup analysis for the association between RC and risk of diabetes.

Subgroup analyses and interaction tests were conducted to explore the relationship between RC levels and the risks of CHD and diabetes. **Figures 7** and **8** illustrate the varying associations observed across different subgroups. The relationship between RC and the risk of CHD and diabetes showed inconsistent patterns. Specifically, the risk of CHD tended to increase in participants who were nonsmokers and had a BMI >30, whereas the risk of diabetes was elevated in participants aged ≥60, with a BMI ≤30, and who were nonsmokers. Participants with hypertension but without CHD also demonstrated a heightened risk of diabetes. Interaction tests indicated that subgroups stratified by age, BMI, smoking status, hypertension, and diabetes/CHD did not significantly modify the association between RC levels and the risks of CHD and diabetes.

## 4 Discussion

In our current investigation, RC exhibited positive correlations with BMI, FBG, HbA1c, LDL-C, TC, and TG, while showing a negative correlation with HDL-C. We observed a significant positive correlation between RC levels and the prevalence of CHD, diabetes, and the combination of CHD with diabetes among postmenopausal women in the United States. After adjusting for potential covariates, the risk of developing CHD, diabetes, and CHD combined with diabetes increased across baseline RC quartiles. Compared with participants in the lowest RC, those in the highest quartile showed a greater incidence of CHD [OR=1.76, 95%CI (1.04, 2.98)], diabetes [OR=1.81, 95%CI (1.30, 2.53)], and CHD combined with diabetes [OR=3.08, 95%CI (1.29, 7.37)] (all P < 0.05). Restricted cubic splines (RCS) curves demonstrated a predominantly linear relationship between RC levels and the risks of CHD, diabetes, and CHD combined with diabetes.

LDL-C remains a cornerstone in current guidelines for preventing CHD. Despite the adoption of high-intensity statin regimens and recent combined therapies with ezetimibe or proprotein convertase subtilisin/kexin type 9 (PCSK9) inhibitors to further lower LDL-C levels, a significant residual risk of CVD persists (25, 26). Notably, statin therapy has been associated with an increased risk of incident diabetes according to the American Heart Association (27), attributed in part to 3-hydroxy-3-methylglutaryl-CoA reductase (HMGCR) inhibition (28). A large-scale meta-analysis of genetic association studies has shown that exposure to LDL-C-lowering genetic variants near NPC1L1 and PCSK9 genes is associated with an increased risk of type 2 diabetes, with a 1.19 to 2.42-fold increase in overall diabetes risk per 1 mmol/L decrease in LDL-C (4). This poses a challenge as efforts continue to aim for lower LDL-C targets. Recent years have seen an increasing focus on RC, supported by numerous observational and genetic studies suggesting that elevated RC is a causal risk factor for CVD (13). For instance, a large cohort study in Korea involving 1,956,452 patients with type 2 diabetes found that those in the highest RC quartile had a 22% higher risk of ischemic stroke and a 28% higher risk of myocardial infarction compared to those in the lowest quartile (29). Epidemiological evidence also underscores that higher RC levels significantly correlate with diabetes development independently of insulin resistance, exacerbating the risk of macrovascular complications among diabetic individuals (30).

While prior studies have explored the association between elevated RC levels and the risks of CHD and diabetes, inconsistencies in RC measurement methods across studies (21) have yielded markedly different plasma RC levels (15). Moreover, no previous studies have concurrently assessed RC in relation to the risks of CHD, diabetes, and their combined outcomes in a homogeneous population, particularly among postmenopausal women experiencing complex physiological and metabolic changes. Our study fills a critical gap in the literature. We observed a significant positive association between RC levels and the prevalence of CHD, diabetes, and their combined occurrence in a cohort of postmenopausal women in the United States. Participants in the highest RC quartile had a 76% higher risk of CHD, an 81% higher risk of diabetes, and a 208% higher risk of CHD combined with diabetes compared to those in the lowest RC quartile.

RC, akin to LDL-C, can infiltrate arterial walls and be absorbed by macrophages to form foam cells (31). However, RC is more readily engulfed by macrophages without oxidation when compared to LDL-C (21, 32). RC has been implicated in promoting atherosclerosis through several mechanisms, including the activation of monocytes, upregulation of pro-inflammatory cytokines, and increased production of thrombotic factors (33, 34). The precise mechanisms linking RC to diabetes risk remain unclear, but insulin resistance and pancreatic β-cell dysfunction likely mediate this association (35). Cholesterol overload has been well-documented to damage pancreatic β-cells. Cholesterol can inhibit insulin secretion through multiple pathways, including oxidative stress-induced apoptosis of pancreatic β-cells (36, 37). Lowering cholesterol has proven beneficial in improving pancreatic β-cell function (38). Compared to LDL-C, RC particles carry a higher cholesterol load, potentially posing greater harm to pancreatic β-cells (39). Elevated RC levels increase free fatty acids (FFAs), promoting changes in pancreatic α-cell insulin signaling and excess glucagon secretion, leading to IR (40). IR and abnormal glucose metabolism can elevate circulating RC levels by affecting its production, metabolism, and clearance (41), thereby exacerbating the “vicious cycle” between insulin resistance and RC levels that heightens the risks of CHD and diabetes.

Postmenopausal women are particularly vulnerable to increased cardiovascular disease risk due to significant alterations in lipid metabolism following estrogen decline, coupled with elevated metabolic indicators such as blood pressure, triglycerides, and waist circumference, along with reduced HDL levels (18, 19, 42). Declining ovarian function and hormonal imbalance promote central visceral fat accumulation and abdominal obesity, correlating with IR in non-adipose tissues and organs, thereby further increasing the risk of diabetes among postmenopausal women (20, 43). Given these complexities, heightened attention and proactive management strategies are warranted for addressing RC-related health risks in postmenopausal women. Current interventions targeting RC include lifestyle modifications such as smoking cessation, moderate alcohol consumption, weight management, and dietary adjustments aimed at reducing saturated fats and increasing physical activity (21). Certain foods and medications, including omega-3 fatty acids, fish oil, liraglutide, and peroxisome proliferator-activated receptor α (PPARα) agonists, have shown promise in reducing RC levels (15, 21). Emerging therapies like inhibition of apolipoprotein C-III (apoC-III) and angiopoietin-related protein 3 (ANGPTL3) also hold potential for lowering circulating RC levels (44–46). Certain plants have also demonstrated notable benefits in combating metabolic diseases (47, 48).

In summary, the health risks associated with elevated RC levels in postmenopausal women necessitate vigilant attention and proactive management strategies. RC not only signifies disease predisposition and progression but also represents a promising therapeutic target for future interventions.

While our study benefited from standardized data collection methods in NHANES to minimize measurement bias, several limitations warrant acknowledgment. The observational nature of our study precludes establishing causal relationships. Despite adjusting for potential confounders, residual confounding from unmeasured variables may influence our findings. Moreover, our study sample was restricted to participants from the United States, necessitating caution in generalizing our findings to other populations.

## 5 Conclusion

Our study reveals a significant positive correlation between RC levels and the prevalence of CHD, diabetes, and the combined risk of CHD with diabetes among postmenopausal women. This study contributes to the growing body of evidence supporting remnant cholesterol as a valuable predictor of cardiovascular and metabolic risks in postmenopausal women. Understanding these associations could potentially inform targeted prevention and management strategies tailored to this vulnerable population.

## Data Availability

Publicly available datasets were analyzed in this study. This data can be found here: The data analyzed in the current study were publicly available and can be found at https://www.cdc.gov/nchs/nhanes/.
